# The Efficacy and Safety of Outpatient Exercise Training for Patients with Chronic Thromboembolic Pulmonary Hypertension After Balloon Pulmonary Angioplasty

**DOI:** 10.3390/jcdd12060216

**Published:** 2025-06-07

**Authors:** Takayuki Masuda, Keitaro Akita, Ryota Sato, Takenori Ikoma, Yusuke Mizuno, Terumori Satoh, Masashi Takao, Kenichiro Suwa, Mikihiro Shimizu, Keiichi Odagiri, Katsuya Yamauchi, Yuichiro Maekawa

**Affiliations:** 1 Department of Rehabilitation Medicine, Hamamatsu University Hospital, 1-20-1 Handayama, Chuo-ku, Hamamatsu 431-3192, Japan; tmasuda@hama-med.ac.jp (T.M.); yamakatu@hama-med.ac.jp (K.Y.); 2 Division of Cardiology, Internal Medicine III, Hamamatsu University School of Medicine, 1-20-1 Handayama, Chuo-ku, Hamamatsu 431-3192, Japan; keitaro.akita@gmail.com (K.A.); rsato.hamamatsu@gmail.com (R.S.); winkoma.cardio.801@gmail.com (T.I.); myusuke@hama-med.ac.jp (Y.M.); tesato@hama-med.ac.jp (T.S.); k-suwa@hama-med.ac.jp (K.S.); 3 Center for Clinical Research, Hamamatsu University Hospital, 1-20-1 Handayama, Chuo-ku, Hamamatsu 431-3192, Japan; shimizu.mi@hama-med.ac.jp (M.S.); kodagiri@hama-med.ac.jp (K.O.)

**Keywords:** rehabilitation, exercise training, gait performance, quality of life

## Abstract

Background: To evaluate the efficacy and safety of outpatient exercise training in clinically stabilized patients with chronic thromboembolic pulmonary hypertension (CTEPH) after balloon pulmonary angioplasty (BPA). Methods: Twenty-four patients with CTEPH after BPA were enrolled in this prospective single-center study. Patients were assigned to the exercise and control groups. The exercise group comprised 12 patients who received 15 weeks of exercise training, with usual care. The control group received only the usual care, without exercise training. The exercise program included aerobic exercise thrice weekly and resistance exercise once or twice weekly. The assessments employed included a 6-min walk test, cardiopulmonary exercise testing, and an emPHasis-10 questionnaire. Results: In the exercise group, the 6-min walk distance was significantly longer (510.0 [467.5, 595.0] m vs. 425.0 [395.0, 465.0] m, *p* = 0.020), the time taken to walk 10 m was shorter (6.4 [5.9, 7.5] s vs. 8.9 [8.1, 9.1] s, *p* = 0.020), and the walking speed was faster (1.6 [1.3, 1.7] m/s vs. 1.1 [1.1, 1.2] m/s, *p* = 0.020) at 15 weeks compared with the results for the control group. The quality of life tended to improve at 15 weeks compared with that before the exercise training. However, hemodynamics did not change significantly before and after the exercise training, and no fatal arrhythmias or syncope were observed. Conclusions: Exercise training improved gait performance, without any adverse events, in patients with CTEPH after BPA. Therefore, exercise training as an adjunct to medical therapy may be a safe potential therapy for patients with CTEPH after BPA.

## 1. Introduction

Exercise training is an effective adjunct therapy for various diseases [1]. Its primary benefits include improvements in exercise tolerance and muscle strength. Specifically, in cases of heart failure, exercise training can cause reductions in total mortality and hospitalization rates, thereby contributing to disease management [2]. Supervised exercise training is beneficial for patients with physically deconditioned pulmonary arterial hypertension (PAH) under medical therapy [3,4,5]. This rehabilitation has also improved physical activities in patients with inoperable chronic thromboembolic pulmonary hypertension (CTEPH) [6]. In addition, in-hospital and home-based rehabilitation is a prerequisite for hemodynamic stability under optimal pharmacotherapy. Previous studies on exercise training for patients with pulmonary hypertension were predominantly focused on PAH cases, with the occasional inclusion of CTEPH cases. CTEPH cases represent only 10–30% of the total research cases [7,8], and comprehensive evidence on exercise training for CTEPH cases is still lacking [9].

Balloon pulmonary angioplasty (BPA) is an effective treatment for improving hemodynamics in patients with CTEPH [10,11,12,13,14]. However, it is reportedly insufficient to improve exercise tolerance [15]. The improvement in quality of life (QOL) depends more on the improvement in the 6-min walk distance (6MWD) than on hemodynamics [12], underscoring the importance of implementing exercise training after BPA-initiated clinical stabilization [3,6]. Exercise training for patients with CTEPH should be conducted in facilities with appropriate experience in patient care and rehabilitation. However, such facilities are not always accessible, even in modern medical practice. Indeed, specialized institutions providing exercise training for patients with CTEPH are limited. The importance of unsupervised outpatient exercise was featured during the coronavirus disease 2019 (COVID-19) pandemic [16,17]. Therefore, the benefit of unsupervised exercise may be applied to patients with CTEPH after BPA.

Thus, this study was performed to clarify the safety and efficacy of exercise training combining supervised outpatient exercise training and unsupervised home exercise in patients with CTEPH after BPA.

## 2. Materials and Methods

### 2.1. Study Protocol

**Figure 1:**
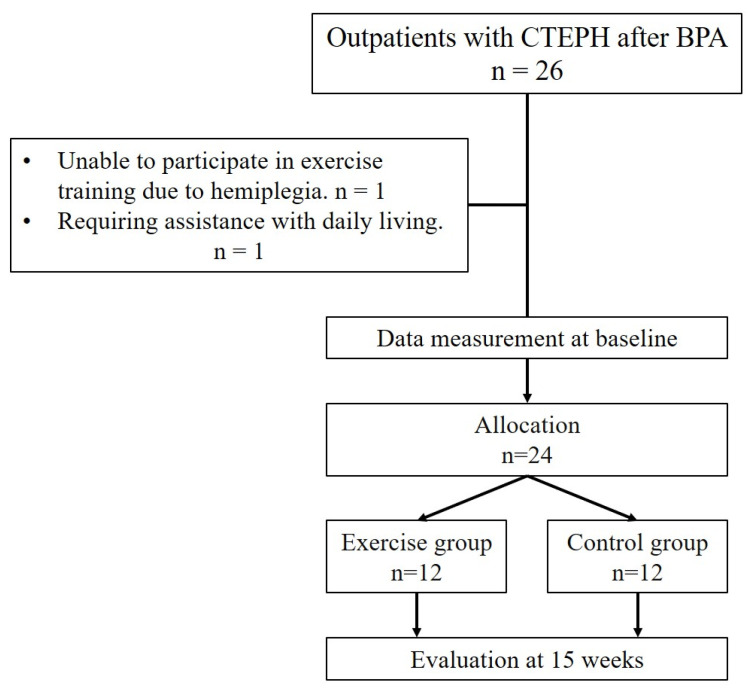
Study flowchart.

This was a single-center, prospective, and non-randomized comparative study. Between June 2019 and July 2023, patients with CTEPH who underwent BPA at Hamamatsu University Hospital were consecutively enrolled (Figure 1). However, those who could not participate in the exercise training program for physical reasons, such as needing assistance with daily living, were excluded. After BPA, all patients were advised to join the designed exercise training program. Those who agreed and did not agree to participate in the exercise training program were assigned to the exercise and control groups, respectively. The exercise group began the 15-week exercise program once hemodynamics stabilized with medical therapy and BPA. However, the control group was observed for the same duration, without exercise training (usual care only). Hemodynamics, physical function, and QOL were evaluated at three time points: before BPA (within 4 weeks before BPA), baseline (within 4 weeks of BPA completion), and follow-up (between 15 and 20 weeks after the baseline).

This study followed the principles of the Declaration of Helsinki, and all participants provided written informed consent. The Hamamatsu University School of Medicine Ethics Committee approved the study (approval number: 19-055).

### 2.2. BPA Procedure

The BPA procedure was performed using the right internal jugular vein. The BPA procedure has been previously described in detail [18]. After a selective pulmonary angiography was performed to confirm the location of the lesion, a 0.014 guide wire (B-Pahm; JAPAN LIFELINE Co., Ltd., Tokyo, Japan) was passed through the lesion. The lesion was dilated in a stepwise fashion through multiple procedures to reduce the risk of pulmonary vascular injury. The lesions with relatively smaller vessels (2.0–3.0 mm diameter) were dilated first, and then as many vessels as possible were treated during the initial BPA procedure. Furthermore, if necessary, lesions were re-dilated with an angiographically appropriate balloon size to optimize the luminal diameter during subsequent BPA procedures. The BPA strategy employed for each period was the same.

### 2.3. Echocardiography, Right Heart Catheterization, and Skeletal Muscle Magnetic Resonance Imaging

The study participants underwent echocardiography (Epiq7; Koninklijke Philips N.V., Amsterdam, The Netherlands). The echocardiography procedure has been previously described in detail [19]. Right heart catheterization (RHC) was performed at baseline and follow-up. The RHC procedure has been previously described in detail [20]. Pulmonary vascular resistance (PVR) and cardiac index were calculated based on Fick measurements of pulmonary flow. Proton magnetic resonance spectroscopy was performed for fat analysis of skeletal muscle. A GE 3T imaging unit with a 32-channel torso array coil (Discovery MR750, GE Healthcare, Waukesha, WI, USA) was used. Echo time, repetition time, number of scans, and number of excitations were set as imaging parameters and were described separately. The region of interest was a 1 cm square cube placed in the left and right soleus muscles.

### 2.4. Primary Endpoint and Secondary Endpoint

The primary endpoint in this study was the 6MWD. The 6MWD has prognostic value in patients with PAH [21]. In a previous study, stratified estimated 1-year mortality was based on the 6MWD (>440 m: low risk, 165 m–440 m: intermediate risk, and <165 m: high risk) [22]. Therefore, the clinically meaningful improvement in 6MWD was defined as >33 m, based on a previous study [23]. The secondary endpoints were mean pulmonary artery pressure (mPAP), anaerobic threshold (AT), peak oxygen uptake (VO_2_), peak work rate (WR), lowest ventilatory equivalent for carbon dioxide (VE/VCO_2_), knee extension strength, gait speed, frailty evaluated using the revised Japanese version of the Cardiovascular Health Study criteria [24], QOL, and adverse events. The degree of frailty was categorized as frail, pre-frail, or robust. The emPHasis-10 score was used to determine the QOL. The emPHasis-10 questionnaire is a pulmonary hypertension-specific, health-related QOL survey [25]. The adverse events included death, worsening World Health Organization-Functional Class (WHO-FC) status, atrial fibrillation, non-sustained ventricular tachycardia, and syncope.

### 2.5. Exercise Training Program

The exercise training program comprised aerobic training thrice weekly and resistance training once or twice weekly. Aerobic activities included a cycle ergometer or walking, with the intensity carefully regulated to stay below the AT, as determined using cardiopulmonary exercise testing (CPET). Each aerobic session lasted 30 min. Resistance training involved bodyweight exercises such as squats and calf raises, performed in three sets of 10–20 repetitions. The exercise training program was divided into supervised sessions during outpatient visits (once or twice weekly) and unsupervised home exercises (once or twice weekly). During supervised outpatient sessions, oxygen saturation (SpO_2_) and electrocardiogram (ECG) results were monitored during exercise. However, for home exercise, SpO_2_ and pulse rate were monitored. Oxygen dosage was adjusted to maintain SpO_2_ levels at >95%. Furthermore, each outpatient visit included a consultation regarding the patient’s home life, with personalized advice provided as necessary. After BPA, the exercise group participated in a 15-week exercise training program. However, the control group underwent usual care for 15 weeks, which included symptom management and adjustments to oral medications.

### 2.6. CPET

CPET was performed using an ergometer (StrengthErgo8, BK-ERG-121, Mitsubishi Electric Engineering Co., Ltd., Tokyo, Japan) and an aero monitor device (AE-310S, Minato Medical Science Co., Ltd., Osaka, Japan). Gas exchange data were recorded using a breath-by-breath system. The CPET protocol included a 5 min rest phase, a 4 min unloaded phase, an incremental phase, and a 3 min recovery phase. Participants were instructed to pedal at 50–55 rpm during the unloaded and incremental phases. The ramp load increment during the incremental phase was tailored to the participant’s physical function and set at 5, 10, or 15 W/min. However, upon reaching symptom limitation during the incremental phase, participants were transitioned to the recovery phase. The test could be terminated at any point by the participant or the examiner if excessive fatigue, respiratory distress, chest discomfort, other discomforts, or ECG abnormalities were observed. AT was determined using the V-slope and trend methods.

### 2.7. Statistical Analysis

Data are presented as a percentage for categorical variables, a mean ± standard deviation (SD) for normally distributed continuous variables, and a median (interquartile range) for non-normally distributed continuous variables. The Shapiro–Wilk test was used to determine whether the data were normally distributed, and the two-sample and paired *t*-tests were used as appropriate. Continuous variables with non-normal distribution were compared using Mann–Whitney U and Wilcoxon signed-rank tests, as appropriate. However, categorical variables were compared using Fisher’s exact test. The Wilcoxon rank sum test was performed to compare each variable in the CPET, walking test, body composition, and skeletal muscle magnetic resonance imaging between the baseline and follow-up periods. The calculated *p*-values were adjusted for differences in variables other than the primary and secondary endpoints between the groups at baseline and follow-up using the Benjamini–Hochberg method. Statistical significance was set at a two-tailed *p*-value < 0.05, and data analysis was conducted using SPSS version 29.0 (IBM, Armonk, NY, USA) and EZR (Saitama Medical Center, Jichi Medical University, Saitama, Japan, version 1.63), which is a graphical user interface for R (R Foundation for Statistical Computing, Vienna, Austria, version 4.2.3).

## 3. Results

Overall, 26 patients with CTEPH underwent BPA; however, two patients who required assistance with activities of daily living and were unable to participate in the exercise program were excluded. Finally, 24 patients were enrolled in this study; they were included in the exercise (n = 12) and control (n = 12) groups, respectively. Patients underwent an average of 6.2 (SD 2.8) BPA sessions. The study flowchart is shown in Figure 1.

Table A1 shows the baseline characteristics of all patients before BPA. Notably, 87.5% of the participants were females, and their body mass index was 23.8 ± 4.3 kg/m^2^. Table A2 shows the effect of BPA on the hemodynamics of all patients. The mPAP, PVR, and N-terminal prohormone of the brain natriuretic peptide (NT-proBNP) values significantly decreased after BPA. Furthermore, regarding CPET, the peak VO_2_ increased (14.1 [12.4, 16.1] mL/kg/min vs. 15.6 [13.5, 18.4] mL/kg/min, *p* = 0.006), and the VE/VCO_2_ slope and lowest VE/VCO_2_ value significantly improved (45.6 [39.2, 52.6] vs. 36.9 [32.3, 39.9], *p* < 0.001; 42.6 [39.8, 48.7] vs. 37.7 [32.9, 42.7], *p* < 0.001). Conversely, there was no significant difference in the AT value (8.9 [7.7, 9.4] mL/kg/min vs. 9.1 [7.7, 9.8] mL/kg/min, *p* = 0.948) before and after BPA.

### 3.1. Baseline Characteristics

Table 1 shows the comparison of baseline characteristics between the exercise and control groups. Neither group showed significant differences in regards to baseline characteristics, including age, proportion of females, body mass index, WHO-FC, NT-proBNP value, and medication.

### 3.2. Primary Endpoint

At baseline, there was no significant difference in the 6MWD between the exercise and control groups (440.0 [401.3, 510.0] m vs. 430.0 [348.8, 471.3] m, *p* = 0.991), whereas, the 6MWD was significantly longer in the exercise group than in the control group during follow-up (510.0 [467.5, 595.0] m vs. 425.0 [395.0, 465.0], *p* = 0.020). Therefore, the change in 6MWD from baseline to 15 weeks was greater in the exercise group than in the control group (75.0 [42.5, 101.3] m vs. −10.0 [−20.0, 13.8] m, *p* = 0.020) (Table 2).

There were no significant differences in the mPAP and PVR values between the two groups (Table A3). Furthermore, the AT, peak VO_2_, peak WR, and lowest VE/VCO_2_ values were similar between the two groups; however, faster gait speed and fewer frail patients were observed in the exercise group compared with the control group (Table 2). No adverse events, including death, worsening WHO-FC, and fatal arrhythmia, occurred in the exercise and control groups.

### 3.3. Findings of Skeletal Muscle Magnetic Resonance Imaging and Echocardiography

There were no significant differences in skeletal muscle MRI findings between the two groups at baseline or follow-up. For echocardiographic findings, the exercise group exhibited lower left ventricular end-diastolic diameters and higher left ventricular ejection fractions at baseline. There were no significant differences in echocardiographic findings between the two groups at follow-up (Table A3).

### 3.4. Comparison Between the Baseline and Follow-Up Periods

**Figure 2:**
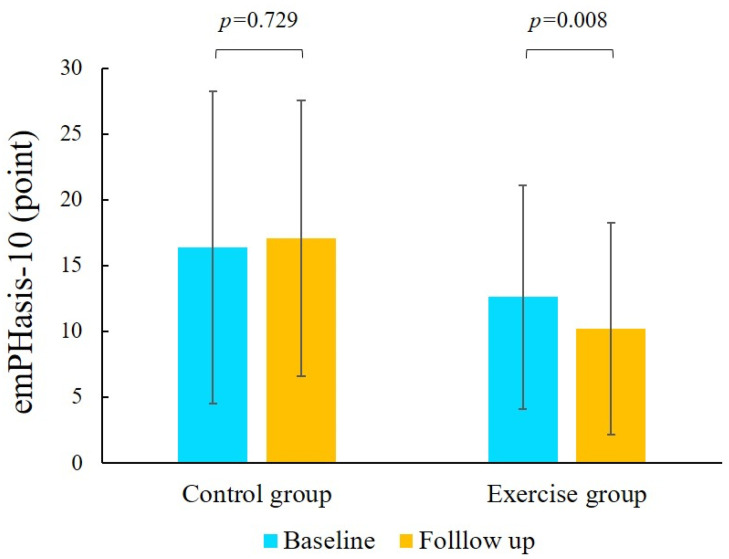
Comparison of emPHasis-10 score in the control and exercise groups between baseline and follow-up.

In the exercise group, peak VO_2_ tended to be higher at follow-up than at baseline. AT and peak WR values at follow-up significantly increased compared with those at baseline. However, in the control group, the lowest VE/VCO_2_ value at follow-up significantly increased compared with that at baseline. In addition, at baseline and follow-up, longer 6MWD, a shorter period of 10 m walking, faster gait speed, and a lower emPHasis-10 score were observed in the exercise group but not in the control group (Figure 2).

## 4. Discussion

Our study results demonstrated a longer 6MWD, shorter period of 10 m walking, and faster gait speed in patients in the exercise group compared with those in the control group. In the exercise group, the emPHasis-10 score was lower at follow-up than at baseline, indicating that the outpatient exercise training program effectively improved QOL after BPA in patients with CTEPH. Furthermore, the outpatient exercise training program decreased the number of frail or pre-frail patients.

Unlike previous studies that initiated exercise training in a hospital setting, in this study, patients were allowed to begin outpatient exercise training and training at home to improve exercise tolerance after BPA. A physiotherapist provided weekly guidance on safe home living and exercises as part of disease management to prevent exacerbation. The primary evaluation index, 6MWD, was longer in the exercise group than in the control group at follow-up. This increase in 6MWD with exercise training was 75.0 [42.5, 101.3] m, surpassing the clinically significant improvement benchmark of 33 m [23]. In addition, a four-stratum risk-assessment tool has been proposed based on refined cut-off levels for WHO-FC, 6MWD, and NT-proBNP, categorizing patients with pulmonary hypertension as low, intermediate–low, intermediate–high, or high risk [22]. According to this tool, after the exercise training program, patients in the exercise group are classified as low risk, whereas those in the control group are categorized as intermediate risk. Consequently, the results may suggest an impact on life expectancy. Therefore, our study results demonstrated that exercise training effectively improved exercise tolerance, even if patients obtained the minimum hemodynamic goal of BPA, which is a final mPAP < 30 mmHg, after BPA [26].

In our study, walking performance and the degree of frailty improved in the exercise group more than that in the control group; however, the CPET parameters were similar between the two groups. The results of a large-scale randomized controlled trial that initiated early stages of exercise training during hospitalization partially supported our findings [27]. Notably, our findings were observed under stable hemodynamics because patients underwent BPA and received guideline-recommended medical therapy. Therefore, improving the CPET parameters was difficult after exercise training. In addition, exercise training further contributed to improvements in the emPHasis-10 scores, suggesting that physical and psychological benefits are sustained in patients with CTEPH after BPA. Therefore, adding the usual care to exercise training in patients with CTEPH after BPA is considered beneficial.

The exercise group tended to have a higher peak VO_2_ at baseline and follow-up than the control group. However, at baseline, VO_2_ at AT and VO_2_/HR tended to be lower, whereas VE/VCO_2_ tended to be higher. These findings were because the exercise group tended to have lower AT and higher ventilatory efficiency (VE/VCO_2_) due to their relatively higher disease severity. On the other hand, peak VO_2_ tended to be higher because the participants in the exercise group were relatively young enough to have had an increase in ventilatory function due to skeletal muscle performance and exercise.

The lowest VE/VCO_2_ value in the control group showed a significant increase after 15 weeks of follow-up; however, it did not increase after 15 weeks of follow-up in the exercise group. This suggests a decrease in ventilatory efficiency and may have indicated a worsening of the condition that was not detectable through resting pulmonary artery pressure. Therefore, the lowest VE/VCO_2_ value can be used to identify CTEPH among patients with chronic thrombi in the pulmonary artery [28]. Patients with CTEPH with a reduced pulmonary vascular bed due to thrombus are likely to exhibit a high resting VE/VCO_2_, and the VE/VCO_2_ slope is expected to be somewhat flat. Conversely, the lowest VE/VCO_2_ value is considered to be high, reflecting an area where blood flow does not increase, even when the alveoli dilate during exercise. Therefore, the lowest VE/VCO_2_ value may be a more accurate reflection of ventilatory efficiency than the VE/VCO_2_ slope for patients with CTEPH. The increase in the lowest VE/VCO_2_ value in the control group is clinically meaningful and may be a basis for recommending participation in exercise training after BPA.

Furthermore, hemodynamics after exercise training were not significantly different between the exercise and control groups, and there was no hemodynamic deterioration due to the exercise training program in this study. Unlike previous studies, we performed a combination of outpatient and home exercises from the early stages of exercise training. Our results suggest that outpatient and home exercise training in hemodynamically stable patients after BPA is effective and safe. Furthermore, our results may suggest one possible solution to the problem of only a few facilities being able to perform supervised rehabilitation of patients with CTEPH.

Previous studies have demonstrated the effectiveness of exercise therapy for PAH; however, there have only been a few reports on its effectiveness for CTEPH [9,29]. This study demonstrated that incorporating home-based exercise therapy into a rehabilitation program (supervised and unsupervised), in addition to outpatient rehabilitation, can improve gait performance and quality of life in patients with stable hemodynamic status after BPA. Additionally, all patients included in this study were treated with BPA in combination with pharmacotherapy, according to the latest guidelines, which may provide useful information for daily clinical practice.

Our study has some limitations. First, this was a single-center, prospective, and non-randomized comparative study, and the number of cases was small. Therefore, there may be a possibility of selection bias. In addition, the sample size was targeted at 20 cases in both groups, based on a previous study [29]. Due to the COVID-19 pandemic and other factors, we could not obtain a sufficient number of samples. Therefore, caution is required when interpreting the findings of this study owing to the small sample size. Second, participation in the exercise group was at the patient’s discretion; therefore, patients who were more enthusiastic about exercising could have joined the exercise group. Third, since the exercises at home are self-reported, we could not confirm to what extent the patients were faithful to the instructions given. However, the 6MWD was longer in the exercise group than in the control group. Fourth, there was a significant difference in gait speed at follow-up between the two groups; however, because the minimally clinically important difference was unknown, it was impossible to determine the extent to which the difference was clinically important. Finally, our follow-up period might not have been long enough to evaluate exercise tolerance, although the follow-up period was consistent with that of a previous randomized control trial [27].

## 5. Conclusions

In conclusion, the outpatient exercise training program, including home rehabilitation, safely improved gait performance and QOL in patients with CTEPH after BPA. Therefore, CTEPH treatment should not end with BPA, and additional exercise training should be considered.

## Figures and Tables

**Table 1:** Baseline characteristics of the exercise and control groups.

	Exercise Group (n = 12)	Control Group (n = 12)	*p*-Value
Clinical characteristics			
Age, years	54.5 ± 15.6	63.2 ± 15.5	0.186
Female, n (%)	11 (91.7)	10 (83.3)	0.500
Body mass index, kg/m^2^	22.8 ± 3.6	24.8 ± 4.9	0.261
WHO-FC II or III or IV n (%)	6 (50.0)	8 (66.7)	0.408
Comorbidities			
Hypertension	0 (0.0)	5 (41.6)	0.019
Dyslipidemia	3 (25.0)	5 (41.6)	0.333
Diabetes	0 (0.0)	1 (8.3)	0.500
Chronic kidney disease	1 (8.3)	3 (25.0)	0.295
Cerebrovascular disease	1 (8.3)	1 (8.3)	0.761
Coronary artery disease	0 (0.0)	1 (8.3)	0.500
History of acute pulmonary embolism	1 (8.3)	2 (16.7)	0.500
Blood test			
NT-proBNP, pg/mL	80.5 [50.3, 113.3]	58.5 [43.5, 67.3]	0.347
Soluble fibrin, μg/mL	1.5 ± 1.7	2.5 ± 1.2	0.132
eGFR, mL/min/1.73 m^2^	72.2 ± 17.7	62.6 ± 15.3	0.169
Medications			
Soluble guanylate cyclase-stimulator, n (%)	12 (100)	12 (100)	1.000
Selective prostacyclin receptor agonist, n (%)	3 (25.0)	0 (0.0)	0.109
Prostacyclin analog, n (%)	1 (8.3)	1 (8.3)	0.761
Warfarin, n (%)	2 (16.7)	4 (33.3)	0.158
DOAC, n (%)	10 (83.3)	8 (66.7)	0.320
Diuretic, n (%)	6 (50.0)	6 (50.0)	0.658

WHO-FC, World Health Organization-Functional Class; NT-proBNP, N-terminal prohormone of brain natriuretic peptide; eGFR, estimated glomerular filtration rate; DOAC, direct oral anticoagulant.

**Table 2:** Comparison of walking test, cardiopulmonary exercise testing, J-CHS categorization, and quality of life between the exercise and control groups at baseline and follow-up.

	Exercise Group (n = 12)		Control Group (n = 12)		Group Difference; *p*-Value		Group Difference; Adjusted *p*-Value	
	Baseline	Follow-Up	Baseline	Follow-Up	Baseline	Follow-Up	Baseline	Follow-Up
Walking test								
6MWD, m	440.0 [401.3, 510.0]	510.0 [467.5, 595.0]	430.0 [348.8, 471.3]	425.0 [395.0, 465.0]	0.574	0.002	0.991	0.020
10 m walk test, s	7.3 [6.9, 8.3]	6.4 [5.9, 7.5]	8.1 [7.4, 9.3]	8.9 [8.1, 9.1]	0.076	0.001	0.716	0.020
Gait speed, m/s	1.4 [1.2, 1.5]	1.6 [1.3, 1.7]	1.2 [1.1, 1.4]	1.1 [1.1, 1.2]	0.076	0.001	0.716	0.020
Change in 6MWD		75.0 [42.5, 101.3]		−10.0 [−20.0, 13.8]		0.002		0.020
Cardiopulmonary exercise testing								
peak VO_2_, mL/kg/min	17.24 [14.62, 19.10]	16.83 [14.94, 19.09]	14.16 [13.04, 15.56]	14.36 [13.87, 16.88]	0.165	0.102	0.784	0.568
AT, mL/kg/min	8.71 [7.70, 10.01]	9.53 [9.25, 11.34]	9.24 [7.65, 9.83]	9.66 [8.41, 9.93]	1.000	0.310	1.000	0.948
WR peak, watt	82.50 [64.25, 90.00]	84.00 [66.00, 100.00]	67.00 [57.75, 85.00]	67.00 [60.00, 82.00]	0.487	0.331	0.991	0.948
VE peak, L/min	49.00 [41.55, 69.28	53.50 [38.30, 75.40]	47.25 [35.95, 55.45]	46.90 [37.10, 56.80]	0.375	0.310	0.991	0.948
VE AT, L/min	18.30 [15.80, 22.27]	23.30 [20.30, 26.10]	20.25 [18.98, 24.08]	21.00 [18.50, 24.90]	0.280	0.757	0.991	0.948
OUES	1111.00 [1003.00, 1338.00]	1084.00 [980.00, 1351.00]	1192.00 [943.75, 1474.75]	1171.00 [1060.75, 1550.50]	0.934	0.637	0.991	0.948
VE/VCO_2_ slope	39.00 [32.95, 40.60]	37.50 [30.20, 42.80]	34.80 [32.80, 37.10]	36.20 [33.00, 38.00]	0.355	0.724	0.991	0.948
Lowest VE/VCO_2_	40.75 [31.27, 43.88]	37.30 [32.10, 42.30]	35.75 [34.28, 39.15]	38.80 [36.20, 41.10]	0.563	0.965	0.991	0.965
VO_2_/WR slope	9.78 [9.18, 10.50]	9.83 [8.47, 9.89]	8.55 [8.10, 9.83]	9.18 [8.59, 10.10]	0.297	0.859	0.991	0.948
Lowest SpO_2_, %	91.00 [88.00, 94.00]	91.50 [88.75, 93.50]	91.00 [89.50, 93.00]	92.50 [90.25, 94.00]	0.928	0.516	0.991	0.948
peak RER	1.21 [1.17, 1.37]	1.21 [1.17, 1.24]	1.25 [1.18, 1.27]	1.24 [1.16, 1.24]	0.938	0.824	0.991	0.948
peak VO_2_/HR	7.11 [5.37, 7.43]	7.33 [5.36, 7.60]	5.68 [5.05, 7.10]	5.80 [5.60, 6.80]	0.700	0.508	0.991	0.948
J-CHS categorization								
Pre-Frail or Frail, n (%)	9 (75.0)	1 (8.3)	8 (66.7)	9 (75.0)	1.000	0.003	1.000	0.023
Quality of life								
emPHasis-10	15.8 ± 11.0	10.2 ± 8.0	16.4 ± 11.9	17.7 ± 10.1	0.891	0.008	0.991	0.052

Adjusted *p*-values throughout this paper have been corrected for multiple testing using the Benjamini–Hochberg correction. J-CHS categorization, Japanese version of the cardiovascular health study criteria categorization.; 6MWD, 6-min walk distance; VO_2_, oxygen uptake; AT, anaerobic threshold; WR, work rate; VE, minute ventilation; OUES, oxygen uptake efficiency slope; VE/VCO_2_ slope, minute ventilation vs. carbon dioxide output slope; VE/VCO_2_, ventilatory equivalent for carbon dioxide; VO_2_/WR slope, oxygen uptake/work rate slope; SpO_2_, oxygen saturation; RER, respiratory exchange ratio; VO_2_/HR, oxygen uptake/heart rate.3.3. Secondary Endpoints

## Data Availability

The deidentified participant data will not be shared due to privacy and ethical restrictions.

## Abbreviations

The following abbreviations are used in this manuscript:

CTEPH	Chronic thromboembolic pulmonary hypertension
BPA	Balloon pulmonary angioplasty
PAH	Pulmonary arterial hypertension
QOL	Quality of life
6MWD	6-min walk distance
COVID-19	Coronavirus disease 2019
RHC	Right heart catheterization
PVR	Pulmonary vascular resistance
mPAP	Mean pulmonary artery pressure
AT	Anaerobic threshold
VO_2_	Oxygen uptake
WR	Work rate
VE/VCO_2_	Ventilatory equivalent for carbon dioxide
WHO-FC	World Health Organization-Functional Class
CPET	Cardiopulmonary exercise testing
SpO_2_	Oxygen saturation
ECG	Electrocardiogram
SD	Standard deviation
NT-proBNP	N-terminal prohormone of brain natriuretic peptide

## Appendix A

**Table A1:** Baseline characteristics of all patients before balloon pulmonary angioplasty.

	Overall Patients, n = 24
Age, years	58.8 ± 15.8
Female, n (%)	21 (87.5)
Body mass index, kg/m^2^	23.8 ± 4.3
Comorbidities	
Hypertension, n (%)	5 (20.8)
Dyslipidemia, n (%)	8 (33.3)
Diabetes, n (%)	1 (4.2)
Chronic kidney disease, n (%)	4 (16.7)
Cerebrovascular disease, n (%)	2 (8.3)
Coronary artery disease, n (%)	1 (4.2)
History of acute pulmonary embolism, n (%)	3 (12.5)
Echocardiography	
LVDd, mm	41.1 ± 5.6
LVDs, mm	25.6 ± 4.0
LVEF, %	67.3 ± 6.1
TRPG, mmHg	54.8 ± 31.9
TAPSE, mm	9.6 ± 8.2
Pulmonary function testing parameters	
FVC, L	2.8 ± 1.0
FEV1, L	2.0 ± 0.7
TLC, L	4.4 ± 1.0
DLCO	14.3 ± 5.8

LVDd, left ventricular end-diastolic diameter; LVDs, left ventricular end-systolic diameter; LVEF, left ventricular ejection fraction; TRPG, tricuspid regurgitation pressure gradient; TAPSE, tricuspid annular plane systolic excursion; FVC, forced vital capacity; FEV1, forced expiratory volume in one; TLC, total lung capacity; DLCO, carbon monoxide diffusing capacity.

**Table A2:** Effect of balloon pulmonary angioplasty in all patients.

	Overall Patients, n = 24		
	Before BPA	After BPA	*p*-Value
Blood test			
NT-proBNP, pg/mL	178 [88.5, 318.5]	65.5 [43.5, 100.5]	<0.001
eGFR, mL/min/1.73 m^2^	66.9 ± 17.2	67.4 ± 16.9	0.823
Echocardiography			
LVDd, mm	41.1 ± 7.1	41.6 ± 4.5	0.664
LVDs, mm	25.3 ± 4.6	25.6 ± 3.9	0.587
LVEF, %	67.5 ± 5.3	67.8 ± 5.3	0.853
TRPG, mmHg	61.6 ± 36.2	28.6 ± 13.3	0.005
TAPSE, mm	8.9 ± 8.6	11.7 ± 8.9	0.466
Right heart catheterization parameters			
mean PAP, mmHg	37.5 ± 10.5	23.9 ± 4.5	<0.001
mean RAP, mmHg	7.9 ± 4.9	3.3 ± 3.0	0.010
mean PAWP, mmHg	9.8 ± 4.3	7.8 ± 3.4	0.192
PVR, wood u	6.6 ± 4.2	3.2 ± 1.4	<0.001
CO, L/min	5.5 ± 2.0	5.7 ± 1.1	0.569
CI, L/min/m^2^	3.4 ± 1.0	3.6 ± 0.6	0.494
Pulmonary function testing parameters			
FVC, L	2.8 ± 0.9	2.9 ± 0.8	0.027
FEV1, L	2.2 ± 0.8	2.3 ± 0.7	0.043
TLC, L	4.3 ± 0.9	4.5 ± 0.9	0.002
DLCO, mL/min/mmHg	14.2 ± 5.4	14.7 ± 5.5	0.144
Medications			
Soluble guanylate cyclase-stimulator, n (%)	24 (100)	24 (100)	1.000
Selective prostacyclin receptor agonist, n (%)	1 (4.2)	3 (12.5)	0.500
Prostacyclin analog, n (%)	2 (8.3)	2 (8.3)	1.000
Warfarin, n (%)	6 (25.0)	5 (20.8)	1.000
DOAC, n (%)	18 (75.0)	19 (79.1)	1.000
Cardiopulmonary exercise testing			
peak VO_2_, mL/kg/min	14.1 [12.4, 16.1]	15.6 [13.5, 18.4]	0.006
VO_2_ AT, mL/kg/min	8.9 [7.7, 9.4]	9.1 [7.7, 9.8]	0.948
WR peak, watt	67.0 [52.5, 80.0]	76.5 [58.3, 88.8]	0.011
VE peak, L/min	50.1 [36.0, 59.1]	48.1 [38.2, 59.2]	0.794
VE AT, L/min	23.2 [18.2, 26.2]	19.3 [16.9, 24.1]	0.231
OUES	1105.0 [884.0, 1233.0]	1111.0 [995.5, 1451.5]	0.049
VE/VCO_2_ slope	45.6 [39.2, 52.6]	36.9 [32.3, 39.9]	<0.001
Lowest VE/VCO_2_	42.6 [39.8, 48.7]	37.7 [32.9, 42.7]	<0.001
VO_2_/WR slope	8.9 [8.0, 10.3]	9.6 [8.3, 10.5]	0.407
Lowest SpO_2_, %	88.5 [85.8, 92.3]	91.0 [89.3, 93.8]	0.005
Quality of life			
emPHasis-10	22.5 ± 9.5	14.5 ± 10.4	0.009

NT-proBNP, N-terminal prohormone of brain natriuretic peptide; eGFR, estimated glomerular filtration rate; LVDd, left ventricular end-diastolic diameter; LVDs, left ventricular end-systolic diameter; LVEF, left ventricular ejection fraction; TRPG, tricuspid regurgitation pressure gradient; TAPSE, tricuspid annular plane systolic excursion; PAP, pulmonary artery pressure; RAP, right atrial pressure; PAWP, pulmonary artery wedge pressure; PVR, pulmonary vascular resistance; CO, cardiac output; CI, cardiac index; FVC, forced vital capacity; FEV1, forced expiratory volume in one; TLC, total lung capacity; DLCO, carbon monoxide diffusing capacity; VO_2_, oxygen uptake; AT, anaerobic threshold; WR, work rate; VE, minute ventilation; OUES, oxygen uptake efficiency slope; VE/VCO_2_ slope, minute ventilation vs. carbon dioxide output slope; VE/VCO_2_, ventilatory equivalent for carbon dioxide; VO_2_/WR slope, oxygen uptake/work rate slope; SpO_2_, oxygen saturation.

**Table A3:** Comparison of right heart catheterization, echocardiographic findings, muscle strength, body composition, and magnetic resonance imaging of the skeletal muscle between the exercise and control groups at baseline and follow-up.

	Exercise Group (n = 12)		Control Group (n = 12)		Group Difference; *p*-Value		Group Difference; Adjusted *p*-Value	
	Baseline	Follow-Up	Baseline	Follow-Up	Baseline	Follow-Up	Baseline	Follow-Up
Right heart catheterization								
mean PAP, mmHg	24.1 ± 5.6	24.9 ± 7.4	23.8 ± 3.3	23.0 ± 4.6	0.860	0.455	0.991	0.948
mean RAP, mmHg	2.57 ± 2.4	4.8 ± 1.8	3.9 ± 3.4	4.1 ± 2.9	0.403	0.492	0.991	0.948
mean PAWP, mmHg	6.3 ± 2.8	10.3 ± 6.0	8.9 ± 3.6	9.5 ± 5.6	0.132	0.729	0.717	0.948
PVR, wood u	4.0 ± 1.9	3.1 ± 1.8	2.7 ± 0.9	3.0 ± 0.7	0.109	0.804	0.716	0.948
CO, L/min	5.8 ± 1.1	5.1 ± 1.5	5.6 ± 1.1	4.6 ± 0.9	0.743	0.296	0.991	0.948
CI, L/min/m^2^	3.6 ± 0.3	3.2 ± 0.7	3.6 ± 0.7	3.0 ± 0.5	0.933	0.381	0.991	0.948
Echocardiographic findings								
LVDd, mm	38.5 ± 3.4	43.6 ± 4.6	43.3 ± 4.1	44.5 ± 6.2	0.045	0.727	0.716	0.948
LVDs, mm	23.4 ± 4.0	27.1 ± 3.9	26.8 ± 3.4	27.5 ± 4.6	0.113	0.845	0.716	0.948
LVEF, %	71.9 ± 4.2	68.3 ± 4.5	65.5 ± 4.6	67.9 ± 4.7	0.025	0.847	0.716	0.948
TRPG, mmHg	30.0 ± 16.2	25.7 ± 14.6	26.5 ± 12.4	27.2 ± 8.6	0.684	0.790	0.991	0.948
TAPSE, mm	9.1 ± 10.1	14.3 ± 9.3	13.5 ± 7.7	13.5 ± 7.6	0.375	0.819	0.991	0.948
Muscle strength								
Knee extension Rt, Nm	31.4 ± 9.8	37.6 ± 10.6	34.3 ± 14.3	35.1 ± 17.7	0.585	0.701	0.991	0.948
Knee extension Lt, Nm	31.0 ± 10.6	36.5 ± 9.1	33.3 ± 16.5	35.5 ± 17.5	0.703	0.875	0.991	0.948
Hand grip Rt, kg	25.5 ± 6.1	27.9 ± 5.1	25.8 ± 7.2	27.7 ± 7.3	0.919	0.933	0.991	0.958
Hand grip Lt, kg	23.0 ± 5.5	25.6 ± 5.5	22.8 ± 5.6	25.1 ± 6.8	0.939	0.849	0.991	0.948
Body composition								
Skeletal muscle index, kg/m^2^	6.20 [5.85, 7.03]	6.25 [5.60, 7.03]	6.30 [5.73, 7.40]	6.20 [5.50, 7.25]	0.692	0.902	0.991	0.951
Skeletal muscle mass, kg	20.70 [18.85, 22.48]	20.55 [18.95, 23.52]	19.05 [16.75, 23.30]	17.80 [16.45, 24.50]	0.575	0.498	0.991	0.948
Body fat mass, kg	17.90 [15.68, 20.33]	18.30 [16.23, 19.35]	26.40 [15.30, 29.50]	20.60 [14.35, 26.05]	0.477	0.805	0.991	0.948
Percent body fat, %	32.20 [30.68, 34.05]	32.10 [30.45, 34.97]	35.10 [29.58, 40.80]	32.90 [29.15, 39.30]	0.468	0.689	0.991	0.948
Magnetic resonance imaging of the skeletal muscle								
IMCL, mmol/kg	13.30 [6.18, 16.74]	9.86 [7.84, 13.51]	6.88 [1.34, 10.84]	5.67 [3.73, 14.24]	0.205	0.481	0.866	0.948
EMCL, mmol/kg	16.56 [12.30, 19.94]	14.72 [9.66, 24.15]	23.47 [8.67, 27.24]	26.57 [11.16, 34.59]	0.841	0.181	0.991	0.882

Adjusted *p*-values throughout this paper have been corrected for multiple testing using the Benjamini–Hochberg correction. PAP, pulmonary artery pressure; RAP, right atrial pressure; PAWP, pulmonary artery wedge pressure; PVR, pulmonary vascular resistance; CO, cardiac output; CI, cardiac index; LVDd, left ventricular end-diastolic diameter; LVDs, left ventricular end-systolic diameter; LVEF, left ventricular ejection fraction; TRPG, tricuspid regurgitation pressure gradient; TAPSE, tricuspid annular plane systolic excursion; IMCL, intramyocellular lipid; EMCL, extramyocellular lipid.
